# Expanding the scope and scale of microbiome research

**DOI:** 10.1186/s13059-019-1804-2

**Published:** 2019-09-05

**Authors:** Rob Knight, Ruth E. Ley, Jeroen Raes, Elizabeth A. Grice

**Affiliations:** 1 Department of Pediatrics, University of California, Gilman Drive, La Jolla, San Diego, CA 92093 USA; 2 Center for Microbiome Innovation, Jacobs School of Engineering, University of California, Gilman Drive, La Jolla, San Diego, CA 92093-0436 USA; 3 Department of Computer Science and Engineering, University of California, Gilman Drive, La Jolla, San Diego, CA 92093-0404 USA; 4 Department of Bioengineering, University of California, La Jolla, San Diego, CA 92093-0412 USA; 5 Department of Microbiome Science, Max Planck Institute for Developmental Biology, Max Planck Ring, 72076 Tübingen, Germany; 6 Laboratory of Molecular Bacteriology, Department of Microbiology and Immunology, Rega Institute, KU Leuven, Herestraat, 3000 Leuven, Belgium; 7 VIB-KU Leuven Center for Microbiology, Campus Gasthuisberg, Rega Instituut, Herestraat, 3000 Leuven, Belgium; 8 Department of Dermatology and Microbiology, Perelman School of Medicine, University of Pennsylvania, Philadelphia, PA 19104 USA

Although the draft human genome was completed in 2001 [1, 2], only in 2006 [3] did we come to realize that the vast majority of the genes in our own species, or any species, remained uncharacterized in ‘genome projects’. Indeed, our understanding of the roles played by complex microbial communities, often termed ‘microbiomes’, in the human body and in the environment continues to expand dramatically.

This Special Issue, published jointly by *Genome Biology* and *BMC Biology*, is a timely exploration and expansion both of our knowledge of microbiomes in different systems and of ways to study microbiomes more effectively. It builds on *Genome Biology*’s nearly decade-long tradition of publishing cutting-edge microbiome research and techniques, and on the journal’s 20-year tradition of publishing sweeping analyses of genomic features across broad swathes of the tree of life.

Many of the articles in this Special Issue focus on new ways to acquire information from DNA sequences obtained using high-throughput sequencing approaches. For example, Fukuyama [4] introduce a new framework for calculating phylogenetic distances that emphasizes deep or shallow branches within the phylogenetic tree, offering different pictures of relationships among microbiomes depending on whether recent or ancient differences are more relevant to the response to a given biological driving factor. Zhu et al. [5] demonstrate that analysis of ‘unmapped reads’, that is, DNA sequences from shotgun metagenomics that don’t match any known genome, can substantially improve our ability to classify samples according to whether they are healthy or diseased, reinforcing the fact that an increased number of complete genome databases for microbes are urgently needed in order to ‘rescue’ these sequences and determine these functions [6]. An alternative approach is to avoid the difficult problem of identifying assembled sequences from short-read data, and to instead associate features of the assembly graph with the host phenotype [7]. Together, these tools will substantially assist efforts to characterize the microbial unknown, and to verify that this characterization is correct.

Several articles in the Special Issue go beyond short-read sequencing. The combination of long-read sequencing and proximity ligation allows the identification of antimicrobial genes and the detection of the plasmids and chromosomes that they are associated with [8]. This approach improves our ability to find pathways of antimicrobial resistance transfer and to determine when plasmids escape their original bacterial host [8]. A readout of the RNA from metatranscriptomic data, rather than just the DNA sequences, has been integral to the discovery of a diverse antibiotic resistance gene reservoir in birds [9]. Similarly, the combination of DNA-based and culture-based approaches was important for the identification of new members of the *Bifidobacteria* in the gut [10], substantially expanding what we know about this key clade of bacteria.

Many important questions about the human microbiome remain unaddressed, especially those relating to the dynamics of the microbiome. The field as a whole is moving from observational studies towards intervention studies, in which temporally resolved study designs offer considerably more power to detect and understand changes [11]. Bouslimani et al. [12] demonstrate the highly personalized dynamic nature of the skin microbiome and metabolome, and show that both the microbial and chemical repertoire of the skin can be changed radically by changing skin-care products.

Moving beyond a parochial view of our own species, Rhoades et al. [13] provide the highest-resolution view yet of the development of the microbiome of infant Rhesus macaques, an important model animal for biomedical research, whereas Malmuthuge et al. [14] provide a high-resolution view of the developing rumen microbiome.

Complex microbial communities are by no means limited to those associated with hosts. This Special Issue also contains papers addressing important questions in the soil microbiome, including the distribution of arsenic resistance genes [15]. These studies reinforce the growing importance of considering soil, root, leaf, seed, and endophytic communities in agriculture, and examine the role of such studies in expanding the food supply for Earth’s growing human population in a sustainable way.

Taken together, several themes emerge. First, even taking into account the advances in this Special Issue, there is still a critical need for the development of new tools. Second, studies that integrate many individual datasets are increasingly important for progress, and tools and resources that enable such meta-analyses will see increasing use [16, 17]. Third, much of the microbial world remains unknown, and we are just starting to scratch the surface of the genomes and functions contained in many ecosystems, especially when we move beyond DNA to consider the transcripts, proteins and molecules in a given ecosystem. Fourth, we need to know much more about the dynamics and spatial structures of microbiomes on scales ranging from our bodies to the entire planet. We can expect that microbiome research will continue to grow at a rapid pace as methods become easier to apply and less expensive, and as more efficient use is made of the vast resources of existing knowledge. Although it has become a cliché that every year is the best year yet for microbiome research, it is a cliché because it is true. The papers in this Special Issue represent both this progress and the potential for yet more advances in the near future. Additional methods, research, and review articles for the Special Issue are still in the pipeline and will continue to be added to the collection.
